# Vitamin D supplementation effects on the clinical outcomes of patients with coronary artery disease: a systematic review and meta-analysis

**DOI:** 10.1038/s41598-020-69762-w

**Published:** 2020-07-31

**Authors:** Leila Sadat Bahrami, Golnaz Ranjbar, Abdolreza Norouzy, Seyyed Mostafa Arabi

**Affiliations:** 1 Department of Nutrition, Faculty of Medicine, Metabolic Syndrome Research Center, Mashhad University of Medical Sciences, Mashhad, Iran; 2 Department of Basic Medical Sciences, Neyshabur University of Medical Sciences, Neyshabur, Iran

**Keywords:** Interventional cardiology, Nutrition

## Abstract

In this systematic review and meta-analysis our aim was to assess the effect of vitamin D supplementation on cardiac outcomes in patients with coronary artery disease (CAD). The search terms were performed from January 2000 to January 2018, only randomized clinical trials (RCT) in human subjects were considered, with no language restrictions. The electronic databases used in this study were: PubMed; Cochran library; Embase; and Scopus. Two independent expert reviewers carried out data extraction according to Cochrane recommendations. Only four RCTs were found in relation to the effects of vitamin D supplementation on the coronary artery disease. In these 299 patients, vitamin D supplementation had significant favorable effects on Diastolic Blood Pressure (DBP) (− 2.96, *p* = 0.02) and Parathyroid hormone (PTH) (− 4.05, *p* < 0.001). However, it had no significant effects on hs-CRP mean difference (− 0.04, *p* = 0.25), total cholesterol (TC) (− 0.46, *p* = 0.83), triglyceride (TG) (0.68, *p* = 0.89), low-density lipoproteins (LDL) (2.08, *p* = 0.56), and high-density lipoproteins (HDL) (− 2.59, *p* = 0.16). In conclusion, the use of vitamin D was associated with improvements in some cardiac outcomes of CAD patients with vitamin D deficiency. Also, further research is needed to clarify these results.

## Introduction

Coronary artery disease (CAD) is the most common of cardiovascular diseases and remains as one of the main causes of morbidity and mortality in the world^1,2^. Its prevalence is increasing in developed and developing countries, where it imposes a heavy financial burden on societies with different demographic backgrounds^3^. CAD is caused by obstruction in the coronary arteries, resulting in impaired oxygenation in the heart muscle, followed by asymptomatic or symptomatic discomfort with persistent angina pain^4,5^. Among the risk factors associated with CAD, obesity, diabetes, hypertension, and physical inactivity are the most common forms. Also, according to previous studies, vitamin D deficiency could act as a risk factor for CAD^6–8^. The proposed mechanisms for these effects include increased levels of renin and angiotensin II, calcification and smooth muscle proliferation, followed by increased lipid profile and features of metabolic syndrome^4,9,10^.

According to a cohort study was conducted in India, less than 5% of CAD patients were vitamin D sufficient, therefore vitamin D deficiency is believed to be highly prevalent in this disease^11^. Several studies have illustrated the relationship between vitamin D deficiency and coronary artery disease. In most of the studies, vitamin D status were inversely related to coronary artery disease^8,9,12–14^. For example, in a meta-analysis study conducted on cross-sectional studies, low concentrations of the calcidiol have been shown to mark the risk of ischemic heart disease and early death^15^. However, few randomized clinical trial studies have also been carried out in this context, in which they have demonstrated conflicting results^16–18^. In this systematic and meta-analysis study, our aim is to conduct a robust evidence-based effect of vitamin D supplementation on cardiac outcomes in CAD patients with vitamin D deficiency.

## Method

### Research methods

**Figure 1:**
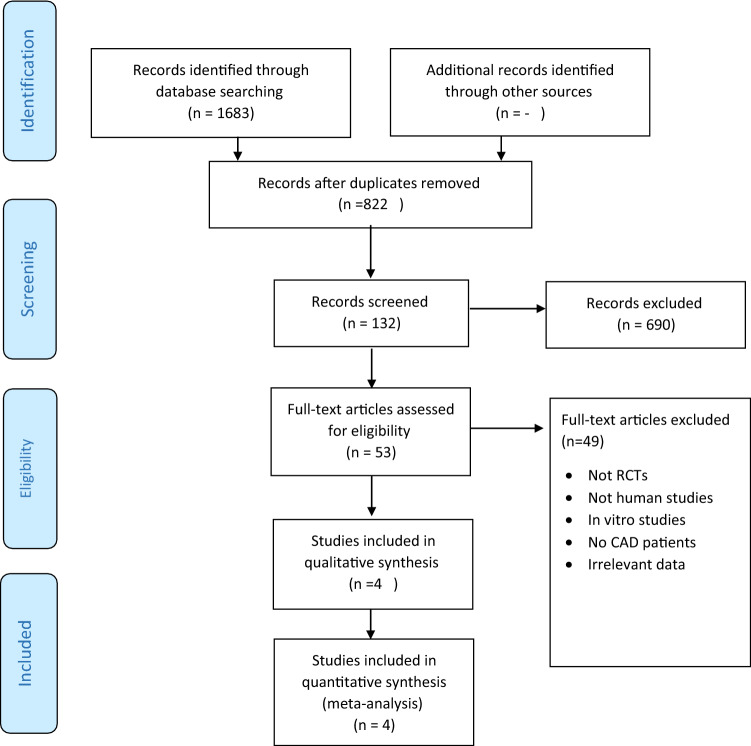
PRISMA flow-diagram of the study selection process.

The Preferred Reporting Items for Systematic Reviews and Meta-analyses guidelines (PRISMA) was considered appropriate for use in this systematic and meta-analysis review as it is covering a public health subject that requires transparent reporting^19^. Thus the effect of vitamin D intervention on clinical and biochemical outcomes in patients with CAD were evaluated by findings of randomized controlled trial (RCT) studies according to PRISMA (details are shown in Fig. 1)^19^.

### Search strategy

We searched PubMed; Cochran library; Embase; and Scopus databases, studies were selected based on inclusion criteria by conducting a comprehensive search using the standard Mesh terms. Search items included vitamin D, vitamin D3, cholecalciferol, ergocalciferol, and calcitriol combined with coronary artery disease, blood pressure, hypertension, cardiovascular, heart disease, coronary disease, inflammation, inflammatory mediators, lipids, total cholesterol, triglycerides, high density lipoprotein (HDL), low density lipoprotein (LDL), high -sensitivity C reactive protein (hs-CRP), parathyroid hormone (PTH), blood pressure and RCTs. Studies search terms were regularly checked in the stated databases from January 2000 to January 2018 for randomized clinical trials in human subjects, with no language restrictions. Moreover, the reference list of each identified article was reviewed and eligible articles (only those reporting RCTs) were also included.

### Study screening and inclusion criteria

By using the PICOS framework (Population, Intervention, Comparison, Outcomes, Study design)^20^, we determined the eligibility of studies and these eligibility criteria are reported in Table 1.

**Table 1:** Inclusion and exclusion criteria of studies.

First author, year (ref)	Inclusion criteria	Exclusion criteria	Methods score according to Cochran collaboration
Sokol et al.^18^	CAD patients with ≥ 50% angiographic stenosis of at least one coronary artery and vitamin D concentrations < 30 ng/ml	GFR < 60 ml/min, liver disease, Hypercalcemia, stage III or IV heart failure, cardiogenic shock, history of gastric or small bowel surgery, pancreatitis, malabsorption, IBD, autoimmune disease, active malignancy, Dilantin, phenobarbital, immunosuppressant therapy, current use of > 800 IU/day of vitamin D	C. Random: yes Blinding: double blind ITT: yes
Zhaoke (2015)	CAD patients with ≥ 50% angiographic stenosis any of the major epicardial coronary arteries, and vitamin D concentrations < 30 ng/ml	Patients did not undergo percutaneous coronary intervention	C. Random: yes Blinding: double blind ITT: yes
Farrokhian et al.^17^	CAD patients with ≥ 50% angiographic stenosis any of the major epicardial coronary arteries, and vitamin D concentrations < 30 ng/ml	Supplementation with vitamin D, myocardial infarction, cardiac surgery within the past 3 months, hepatic failure	C. Random: yes Blinding: double blind ITT: yes
Bahrami et al.^16^	CAD patients with ≥ 50% angiographic stenosis any of the major epicardial coronary arteries, and vitamin D concentrations < 30 ng/ml	BMI > 35 kg/m^2^; cancer, myocardial infarction, liver disease, kidney disorders; consuming vitamin D supplement (oral and/or intravenous) in the previous 4 months; consuming of herbal supplement; the routine intake of vitamin D-fortified foods; pregnancy; lactation; smoking; alcohol consumption	C. Random: yes Blinding: double blind ITT: no

*CAD* coronary artery disease, *GFR* glomerular filtration rate, *IBD* inflammatory bowel disease, *BMI* body mass index, *ITT* intention to treat, *C* computerized.

1. Participants: Adult patients with CAD and no restrictions on sex, age and race.
2. Interventions: Studies with any form and dose of vitamin D supplements.
3. Comparison: vitamin D group with placebo group.
4. Outcomes: Evaluating the concentrations of hs-CRP for inflammation, blood pressure, lipid profile and PTH.
5. Study design: RCTs.

## Exclusion criteria

1. Editorials, case reports, letters to the editor, review articles, and studies conducted on animal subjects.
2. Studies in which diagnosed CAD patients did not consume vitamin D supplements.
3. Studies that included patients without CAD.

### Data extraction

Two independent expert reviewers (AM and BL) carried out the data extraction according to Cochrane recommendations. Included articles were studied for relevance and content, data were extracted under the following headings: name of authors, country, year of publication, and study design; number of participants and demographics; kind of randomization; duration of intervention; type and dose of vitamin D regimen and type of placebo used; outcome description and evaluation; mean values and their standard deviations were obtained for continues variables; and intention-to-treat analysis.

### Risk of bias and quality assessment

**Figure 2:**
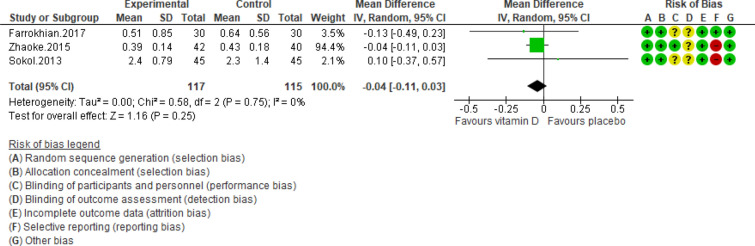
Forest plot of randomized controlled trials showing weighted mean difference in hs-CRP concentrations between the vitamin D-supplemented and placebo groups for all eligible studies. For all the inclusion studies pooled, the non-significant effect of vitamin D supplement on reducing hs-CRP concentrations was observed (*P* value for heterogeneity = 0.75, and χ^2^ = 0.58).

**Figure 3:**
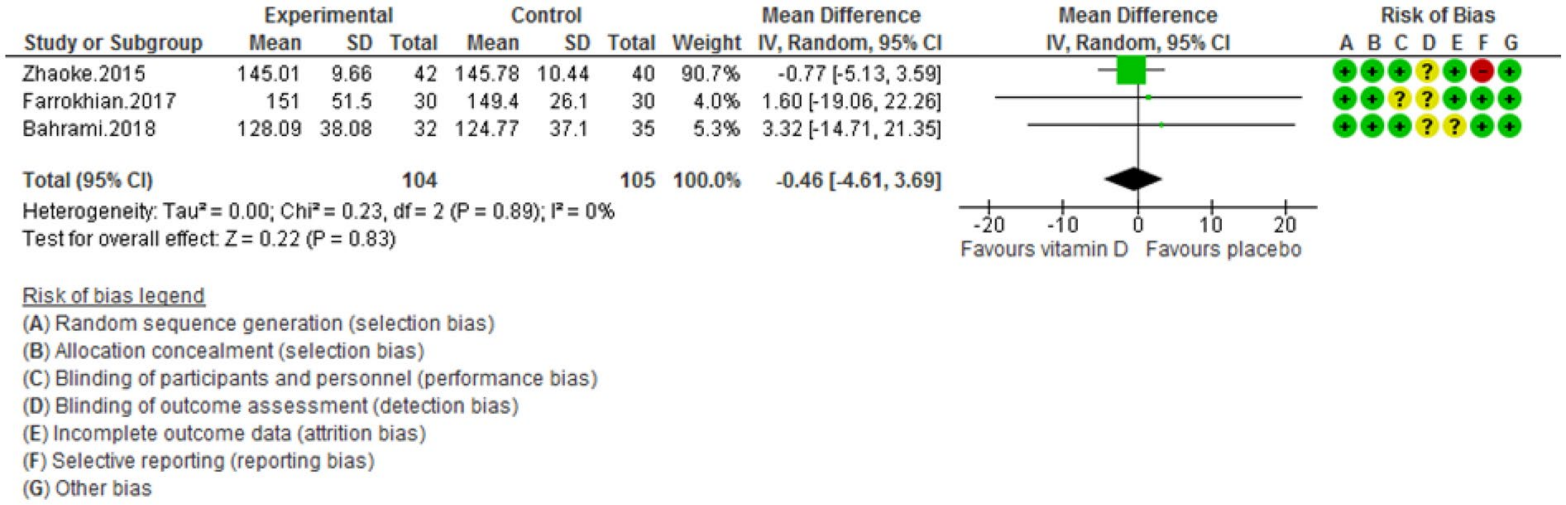
Forest plot of randomized controlled trials showing weighted mean difference in total Cholesterol levels between the vitamin D-supplemented and placebo groups for all eligible studies. For all the inclusion studies pooled, the non-significant effect of vitamin D supplement on reducing total Cholesterol levels was observed (*P* value for heterogeneity = 0.89, and χ^2^ = 0.23).

**Figure 4:**
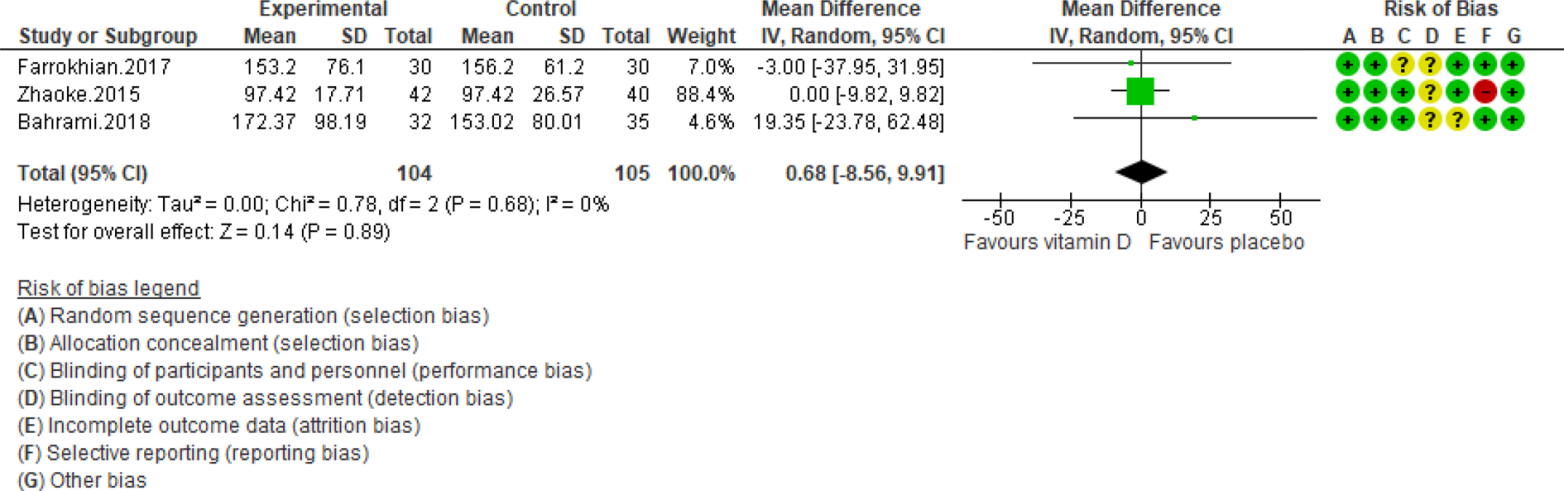
Forest plot of randomized controlled trials showing weighted mean difference in triglyceride levels between the vitamin D-supplemented and placebo groups for all eligible studies. For all the inclusion studies pooled, the non-significant effect of vitamin D supplement on reducing triglyceride levels was observed (*P* value for heterogeneity = 0.68, and χ^2^ = 0.78).

**Figure 5:**
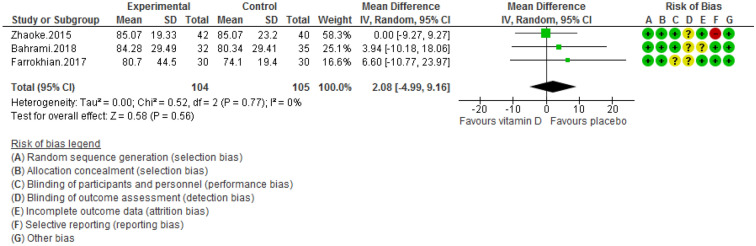
Forest plot of randomized controlled trials showing weighted mean difference in LDL levels between the vitamin D-supplemented and placebo groups for all eligible studies. For all the inclusion studies pooled, the non-significant effect of vitamin D supplement on reducing LDL levels was observed (*P* value for heterogeneity = 0.77, and χ^2^= 0.52).

**Figure 6:**
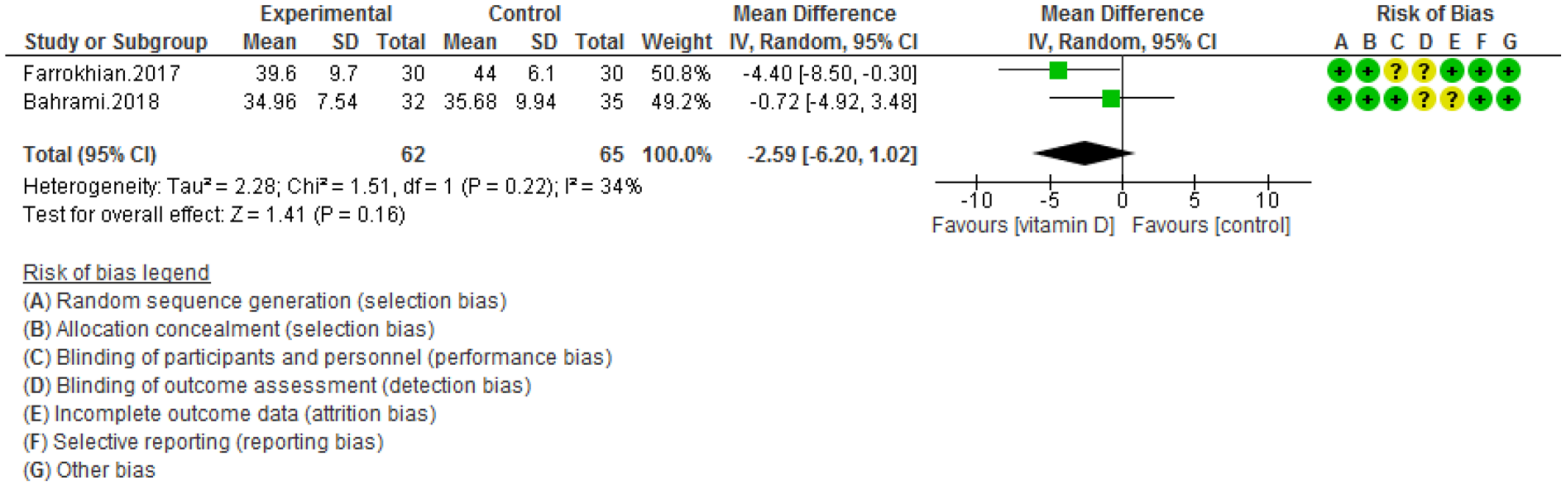
Forest plot of randomized controlled trials showing weighted mean difference in HDL levels between the vitamin D-supplemented and placebo groups for all eligible studies. For all the inclusion studies pooled, the non-significant effect of vitamin D supplement on HDL levels was observed (*P* value for heterogeneity = 0.22, and χ^2^ = 1.51).

**Figure 7:**
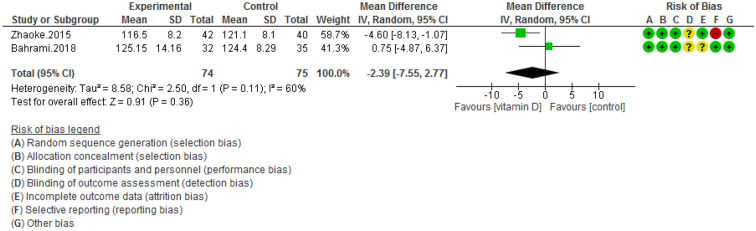
Forest plot of randomized controlled trials showing weighted mean difference in SBP levels between the vitamin D-supplemented and placebo groups for all eligible studies. For all the inclusion studies pooled, the non-significant effect of vitamin D supplement on systolic blood pressure levels was observed (*P* value for heterogeneity = 0.11, and χ^2^ = 2.50).

**Figure 8:**
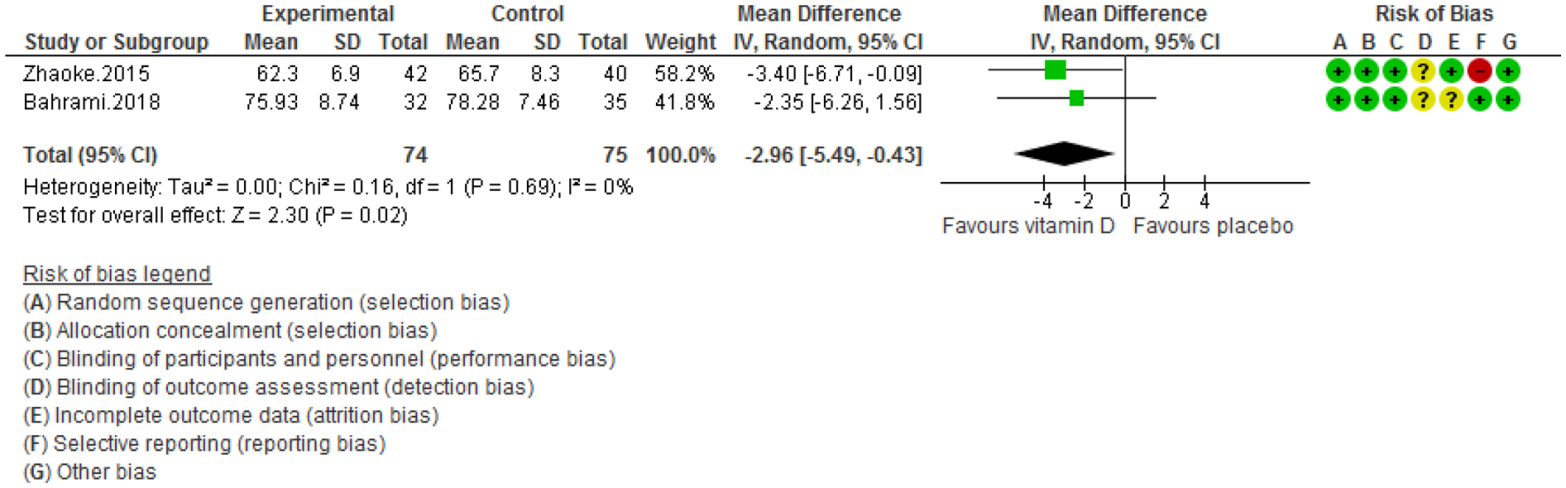
Forest plot of randomized controlled trials showing weighted mean difference in DBP levels between the vitamin D-supplemented and placebo groups for all eligible studies. For all the inclusion studies pooled, the significant effect of vitamin D supplement on reducing diastolic blood pressure levels was observed (*P* value for heterogeneity = 0.69, and χ^2^ = 0.16).

**Figure 9:**
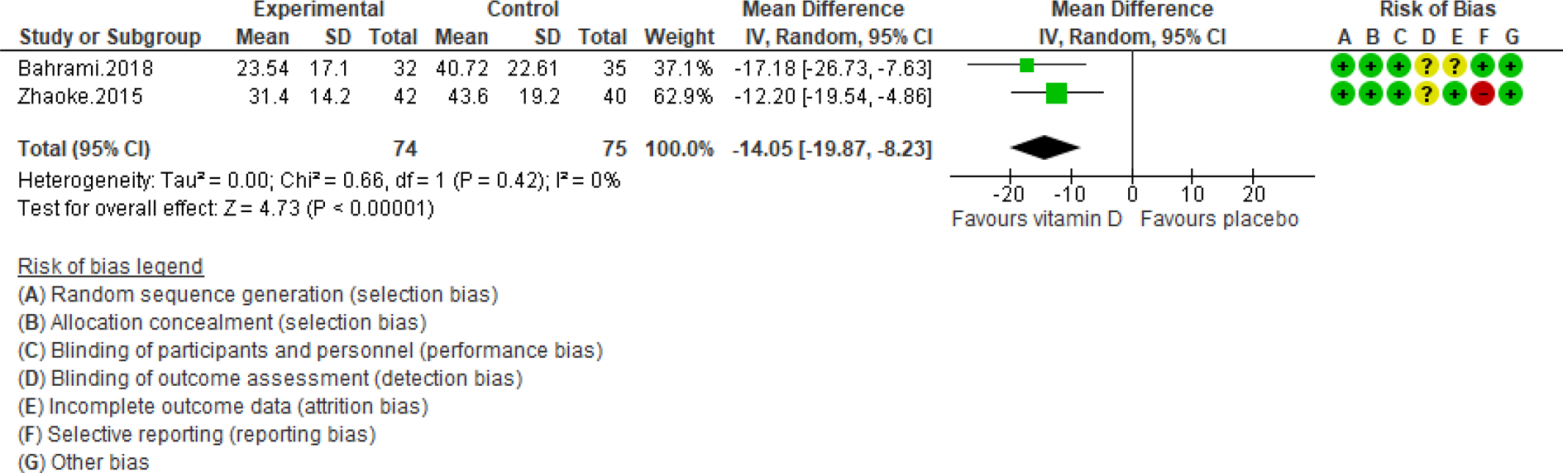
Forest plot of randomized controlled trials showing weighted mean difference in PTH concentrations between the vitamin D-supplemented and placebo groups for all eligible studies. For all the inclusion studies pooled, the significant effect of vitamin D supplement on reducing PTH concentrations was observed (*P* value for heterogeneity = 0.42, and χ^2^ = 0.66).

All RCTs were assessed by two independent reviewers according to Cochrane pre-specified criteria^21^. Using this strategy, each RCT was categorized and rated for bias as high, low and unclear risk of bias. The studies which had at least 3 items for risk of bias were categorized as good quality; studies were categorized as fair with two items for risk of bias; and studies with ≤ 1 item for risk of bias were categorized as poor (details are shown in Figs. 2, 3, 4, 5, 6, 7, 8, 9). Also, the quality of each item was examined by using the method of Cochrane Collaboration risk of bias tools^21^. Reviewers graded quality score of studies by showing the risk of bias less than two a low quality score and higher than two an appropriate quality. Any disagreements were resolved by the third reviewer (AN) (Table 1).

### Statistical analysis

For meta-analysis, collected effect measure after supplementation period were pooled into weight mean difference (WMD) with 95% confidence intervals (CI)^22^. If the variables were non-random in terms of quantity we used them for a fixed model^22^, however if variables heterogeneity (het) existed, the random model was used. When there is heterogeneity that cannot be clarified, one statical approach is to combine it into a random-effects model. This model involves an assumption that the effects being estimated in the different studies are not equal, but accordance some distribution. The center of this distribution explained the mean of the effects, while its width describes the degree of heterogeneity^23^. Heterogeneity was calculated by using the I^2^ ^2^ test with weighted Mantele-Haenszel method, in this regard, I^2^ ^2^ > 50% shows a notable heterogeneity^24^. According to the Egger and Begg statistical tests and visual symmetry of funnel plots, publication bias was determined^25,26^. All the pooled analyses were conducted in Review Manager V.5.3.5 software (Cochrane IMS, Oxford, UK) and publication bias was performed by Comprehensive Meta-Analysis Software V.2. (Biostat, NJ) *P* value < 0.05 was considered statistically significant.

## Results

Selection of studies and screening process are explained in PRISMA flow chart-diagram Fig. 1. A total of 1683 titles peer reviewed publications were retrieved; after scanning the titles, 822 were removed due to duplication and 690 were excluded as they lacked relevance. In the next step, 49 studies were eligible for full-text review. Finally, only four RCT studies met the eligibility criteria for pooled analysis as explained in Fig. 1^16–18,27^.

### Study characteristics

All four RCTs had a parallel design and their intervention period ranged from 8 weeks 16 to 6 months^17,27^. Studies were published online in 2012–2018 and they originated from China^27^, United States^18^ and Iran^16,17^, respectively. The range of sample size was from 60 to 91^17,18,27^ and age of participants were above 50 years old (Table 2).

**Table 2:**
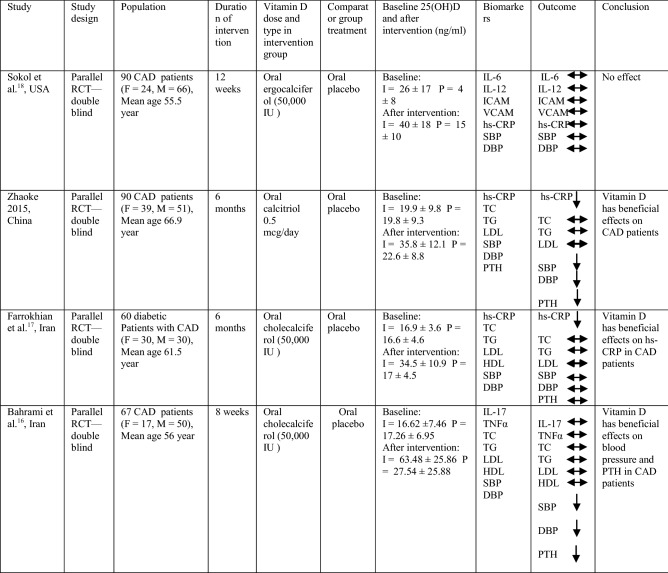
Clinical trials studies evaluating effect of vitamin D supplementation versus placebo in CAD patients.

*F* female, *M* male, *CAD* coronary artery disease, *I* intervention group, *P* placebo group, *IL* interleukin, *ICAM* intercellular adhesion molecule; *VCAM* vascular cell adhesion protein, *hs-CRP* high-sensitivity C-reactive protein, *TNF* tumor necrosis factor, *TC* total cholesterol, *TG* triglyceride, *LDL* low density lipoprotein, *HDL* high density lipoprotein, *SBP* systolic blood pressure, *DBP* diastolic blood pressure, *PTH* parathyroid hormone.

### Participant characteristics

In studies of CAD patients (n = 4 RCTs), the mean age of subjects varied from 55 to 66 years old (Table 2). More than 50% of participants in these 4 RCTs were men. Mean baseline body mass index (BMI) in three studies was ranged from 23.4 to 30.25 kg/m^2^. The range of mean baseline 25(OH) D concentrations was from 16.62 ng/mL to 26 ng/mL, as reported in four RCTs (Table 1)^16,18^.

### Intervention characteristics

Oral tablet of vitamin D was used in all four RCTs, with dose of 0.5 μg daily in one of the RCTs^27^ and weekly dose of 50,000 IU in rest of RCT studies. Of these four RCTs, in two studies participants were supplemented with cholecalciferol^16,17^ and in one study participants were supplemented with ergocalciferol^18^ and in the other one participants were supplemented with oral calcitriol^27^. All of these studies were lasted for between 8 weeks to 6 months as reported in (Table 2).

### Outcome measures

Most studies reported hs-CRP (n = 3 RCTs) as their primary outcomes^17,18,27^, also concentrations of inflammatory cytokines, lipid profiles (n = 3 RCTs)^16,17,27^, blood pressure levels (n = 3 RCTs)^16,17,27^ and PTH status (n = 2 RCTs)^16,27^ were examined as secondary outcomes (Table 2).

### Risk of bias assessment

The risk of bias was evaluated in double-blinded studies and is presented in Figs. 2, 3, 4, 5, 6, 7, 8, and 9. All four trials declared dropout rates^16–18,27^; however, intention to-treat analyses were reported in only three studies^17,18,27^. Reporting bias was detected high risk in two studies^18,27^, while two of them were low risk^16,17^. Finally, the overall quality of each study was evaluated and recognized as “good quality”, since at least two items with low risk of bias were determined in all of these four studies^16–18,27^.

### Meta-analyses

Pooling of three RCTs (n = 235)^17,18,27^ indicated a non-significant difference in hs-CRP concentrations between case and control groups [WMD (95% CI) = − 0.04 [− 0.11, 0.03]; *p* = 0.25; *I*^2^ = 0%; *Phet* = 0.75 (Fig. 2). Data from three RCTs (n = 209)^16,17,27^ revealed no significant difference in TC levels between intervention and placebo groups [WMD (95% CI) − 0.46 [− 4.61, 3.69]; *p* = 0.83; *I*^2^ = 0%; *Phet* = 0.89] (Fig. 3). Pooled data from three RCTs (n = 209)^16,17,27^, showed no considerable difference in TG levels between vitamin D and placebo groups [WMD (95% CI) 0.68 [− 8.56, 9.91]; *p* = 0.89; *I*^2^ = 0%; *Phet* = 0.58 ] (Fig. 4). Similarly, weighted data of three RCTs (n = 154) ^16,17,27^ showed no significant difference in LDL concentrations between two groups [(WMD (95% CI) 2.08 [− 4.99, 9.16]; *p* = 0.56; *I*^2^ = 0%; *Phet* = 0.77 ] (Fig. 5). Also, weighted data of two RCTs (n = 127)^16,27^ indicated no significant difference in HDL levels between the intervention and placebo groups [(WMD (95% CI) − 2.59 [− 6.20, 1.02]; *p* = 0.16; *I*^2^ = 34%; *Phet* = 0.22] (Fig. 6). According to our analysis in total 149 subjects from 2 RCTs^16,27^ there was not a significant difference in Systolic Blood Pressure (SBP) levels between case and control groups [WMD (95% CI) = − 2.39 [− 7.55, 2.77]; *p* = 0.36; *I*^2^ = 60%; *Phet* = 0.11] (Fig. 7) with moderate heterogeneity. Also, pooled analysis of these two studies (n = 149) ^16,27^ indicated a notable difference in Diastolic Blood Pressure (DBP) levels between vitamin D and placebo groups [WMD (95% CI) = − 2.96 [− 5.49, − 0.43]; *p* = 0.02; *I*^2^ = 0%; *Phet* = 0.69] (Fig. 8). Weighted for the data from two RCTs (n = 149)^16,27^ revealed a significant change in PTH concentrations between intervention and placebo groups [WMD (95% CI) − 14.05 [− 19.87, − 8.23]; *p* = 0.00001; *I*^2^ = 0%; *Phet* = 0.42] (Fig. 9).

### Descriptive analyses

In the study by Sokol et al.^18^, 50,000 IU ergocalciferol intake per week for 12 weeks had no significant effect on the concentrations of IL-6 (*p* = 0.94), IL-12 (*p* = 0.72), Intercellular Adhesion Molecule (ICAM) (*p* = 0.048) and vascular cell adhesion molecule (VCAM) (*p* = 0.79). Moreover, in another study carried out by Bahrami et al. 16, 50,000 IU cholecalciferol supplementation per week for 8 weeks did not cause a favorable effect on IL-17 (*p* = 0.585) and TNF-α (*p* = 0.734) levels. In the study conducted by Farrokhian et al.^17^ cholecalciferol supplements intake with dose of 50,000 IU per week for 6 months showed a considerable effect on nitric oxide (NO) levels (*p* < 0.001), Malondialdehyde (MDA) (*p* < 0.001) levels, however it did not induce significant effect on total antioxidant capacity (TAC) levels (*p* = 0.52) in CAD patients.

### Publication bias

According to Egger and Begg statistical tests 25, 26, we found no existed publication bias for hs-CRP (*p* = 0.90; *p* = 0.60), TC (*p* = 0.39; *p* = 0.60), TG (*p* = 0.46; *p* = 0.61) and LDL (*p* = 0.05; *p* = 0.29) levels. Studies reporting on HDL, SBP, DBP and PTH concentrations due to limited quantity (n = 2 RCTs) in this meta-analysis could not be assessed for publication bias.

## Discussion

### Summary of findings

The pooled outcome of this study demonstrated that groups with vitamin D administration showed favorable impacts in diastolic blood pressure and parathyroid hormone levels as compared to placebo groups. However, there was not a significant difference between vitamin D and control groups with regards to levels of hs-CRP, total cholesterol, triglyceride, LDL, HDL and SBP. Our findings suggest that vitamin D supplementation may have a modest clinical effects in CAD patients. Previously, human cross-sectional studies, demonstrated a reverse association between serum vitamin D concentrations and inflammation in heart failure patients. In our meta-analysis with four clinical trials in CAD patients, vitamin D supplements did not reduce the circulating hs-CRP concentrations. While, current study findings are contradictory to a previous meta-analysis by Jiang et al.^28^, where they reported lower concentrations of hs-CRP in treatment group compared to placebo group. However, in support of our study, Rodriguez et al.^29^, reported that pooled outcomes from three studies with 231 heart failure patients illustrated that vitamin D supplementation had no effect on CRP concentrations. The possible mechanism associated with Vitamin D in the regulation of lipid profile levels, could be due to the high lipoprotein lipase activity, increase in calcium absorption rate and decrease in fatty acid absorption levels and LDL formation^30,31^. In the current review, we demonstrated that vitamin D supplementation had no significant effect on the LDL levels in CAD patients. In contrast to our results, according to a meta-analysis study by Mirhosseini et al.^32^, vitamin D treatment in obese subjects improved their lipid profile. In addition, Jafari et al.^33^, also presented a significant decline in the levels of total serum cholesterol, TG and LDL in diabetic patients. This inconsistency between other reported results and the results of our study are probably due to the heterogeneity of the population in Jafari et al.^33^, study and the inclusion of healthy individuals in the study by Mirhosseini et al.^32^.

Notably, antihypertensive function of cholecalciferol is proposed through suppression of the renin angiotensin pathway with its anti-endothelial stiffness effect, followed by secondary hyperparathyroidism prevention. Our meta-analysis demonstrated a significant reduction in DBP levels by cholecalciferol supplementation in CAD patients, which was consistent with previous findings in a study conducted by Mirhosseini^32^, while inconsistent with Beveridge et al.^34^, findings. Moreover, in the current meta-analysis the pooled analysis indicated no effect of SBP reduction in these patients. The moderate heterogeneity for weighted SBP results propose that a clinically significant reduction in blood pressure is unlikely, based on the selected dose of vitamin D in this analysis. Moreover, the moderate heterogeneity for weighted systolic blood pressure suggests no effects of vitamin D supplementation on systolic blood pressure. These outcomes are in accordance with several previous meta-analyses^35–37^. While, it is important to note that different categories of patients, numbers, dose of vitamin D and duration of interventions were pooled in those studies. Since vitamin D deficiency results in parathyroid gland hyperactivity, the PTH concentrations increases^38^. Sudden increase in PTH concentrations, leads to transport of large amount of calcium into the cardiocytes, where heart muscles become hardened^35^. Moreover, the change in the calcium concentrations in the smooth muscle of vessels may lead to muscle contraction and therefore increases the levels of blood pressure in CAD patients. In the present study, vitamin D intervention in CAD patients could suppress the production of PTH levels, in comparison with control group. This result was in accordance with findings from a study conducted by Mirhosseini et al.^32^.

### Limitations of previous studies included

All of the 4 included studies had a small sample size and short duration of interventions. Moreover, smoking status was not reported in most of these studies, which may influence the outcomes of patients with CAD treated with vitamin D supplements.

### Current study limitations and strengths

Due to limited number of studies no meta-regression or subgroup analysis were conducted on the effect of confounding factors on the results of current study. Moreover, different types, doses and durations of vitamin D supplements were used (ergocalciferol and calcitriol), which may lead to some limitations to our analysis. The strengths of the current study include: use of only randomized clinical trial studies with low risk of bias which are considered as the gold standard. A comprehensive search on electronic databases with no language restrictions and no publication bias were conducted in this systematic review and meta-analysis.

## Conclusions

In conclusion, our results indicated that vitamin D supplementation in vitamin D deficient subjects had a favorable effect on diastolic blood pressure levels and parathyroid hormone concentrations in comparison with control group. Therefore, vitamin D may be recommended to be used as an adjunct therapy to routine treatment in coronary artery disease patients with vitamin D deficiency. However, further well-designed clinical trials with on a larger scale and of longer duration are required to determine the actual impact of vitamin D supplementation on clinical outcomes of patients with CAD.
